# Fate of Adipose Progenitor Cells in Obesity-Related Chronic Inflammation

**DOI:** 10.3389/fcell.2020.00644

**Published:** 2020-07-14

**Authors:** Iryna Pyrina, Kyoung-Jin Chung, Zoi Michailidou, Michael Koutsilieris, Triantafyllos Chavakis, Antonios Chatzigeorgiou

**Affiliations:** ^1^Institute for Clinical Chemistry and Laboratory Medicine, University Hospital and Faculty of Medicine Technische Universität Dresden, Dresden, Germany; ^2^Centre for Cardiovascular Science, Queen’s Medical Research Institute, University of Edinburgh, Edinburgh, United Kingdom; ^3^Department of Physiology, Medical School, National and Kapodistrian University of Athens, Athens, Greece; ^4^Paul Langerhans Institute Dresden of the Helmholtz Center Munich, University Hospital and Faculty of Medicine, Technische Universität Dresden, Dresden, Germany

**Keywords:** preadipocyte, adipose progenitor, inflammation, adipogenesis, beiging, obesity, fibrosis

## Abstract

Adipose progenitor cells, or preadipocytes, constitute a small population of immature cells within the adipose tissue. They are a heterogeneous group of cells, in which different subtypes have a varying degree of commitment toward diverse cell fates, contributing to white and beige adipogenesis, fibrosis or maintenance of an immature cell phenotype with proliferation capacity. Mature adipocytes as well as cells of the immune system residing in the adipose tissue can modulate the function and differentiation potential of preadipocytes in a contact- and/or paracrine-dependent manner. In the course of obesity, the accumulation of immune cells within the adipose tissue contributes to the development of a pro-inflammatory microenvironment in the tissue. Under such circumstances, the crosstalk between preadipocytes and immune or parenchymal cells of the adipose tissue may critically regulate the differentiation of preadipocytes into white adipocytes, beige adipocytes, or myofibroblasts, thereby influencing adipose tissue expansion and adipose tissue dysfunction, including downregulation of beige adipogenesis and development of fibrosis. The present review will outline the current knowledge about factors shaping cell fate decisions of adipose progenitor cells in the context of obesity-related inflammation.

## Introduction

In the past two decades, adipose tissue (AT) has been extensively studied in both rodents and humans, especially regarding mechanisms involved in obesity-related metabolic dysregulation. There are two morphologically and functionally distinct types of AT: white AT (WAT) and brown AT (BAT). WAT is predominantly responsible for energy storage in the form of triglycerides and secretes hormonal regulators, namely adipokines, such as leptin and adiponectin, which can regulate whole-body’s metabolic homeostasis. BAT, in contrast, has a non-shivering heat production capacity, due to expression of uncoupling protein-1 (UCP-1) (Kershaw and Flier, 2004; Peirce et al., 2014; Rosen and Spiegelman, 2014). Beige, or brite adipocytes represent a type of adipocytes that morphologically resemble white rather than brown fat cells and reside within WAT, but express UCP-1 and exert brown-like properties (Rosen and Spiegelman, 2014; Alexaki and Chavakis, 2016; Cinti and Giordano, 2020).

In addition to adipocytes, the stromal vascular cell fraction (SVF) of the AT contains further cell types, such as endothelial cells, various immune cells and adipocyte progenitors (preadipocytes or adipose progenitors cells, APs) (Ailhaud et al., 1992; Church et al., 2014). APs reside in perivascular regions of the AT, and can differentiate into mature adipocytes (Tang et al., 2008; Rosen and Spiegelman, 2014; Vishvanath et al., 2016). A recent study defined a developmental hierarchy of APs starting from Dipeptidyl peptidase-4-postive (DPP4^+^) cells that give rise to committed ICAM1^+^ and CD142^+^ preadipocytes capable of adipogenic differentiation (Merrick et al., 2019). Commonly, preadipocytes are described as CD45^–^, CD31^–^, Lin^–^, CD29^+^, and Sca-1^+^ cells (Rodeheffer et al., 2008; Tang et al., 2008; Berry and Rodeheffer, 2013). Platelet-derived growth factor-receptor β positive (PDGFRβ^+^) APs were described as predisposed to a white adipogenic (Gao et al., 2018), while PDGFRα^+^ progenitors to a beige adipogenic or a fibrogenic phenotype (Lee et al., 2012; Marcelin et al., 2017). A recent study provides an insight in the heterogeneity of APs in the mouse VAT based on scRNAseq analysis, demonstrating the existence of two distinct populations of AT-derived stem cells and three populations of preadipocytes (Cho et al., 2019).

During obesity, the expansion of the AT is driven by two processes: hypertrophy (increased adipocyte size) and hyperplasia (increased adipocyte numbers). Hyperplastic growth is considered more metabolically favorable (Shao et al., 2018; Vishvanath and Gupta, 2019), while AT hypertrophy is associated with the development of hypoxia and release of pro-inflammatory cytokines and chemokines by the adipocytes, leading to the recruitment of immune cells and the formation of a pro-inflammatory microenvironment in the AT (Arner et al., 2010; Chatzigeorgiou et al., 2014; García-Martín et al., 2015; Choe et al., 2016; Chung et al., 2018; Michailidou, 2019). Macrophages play a crucial role in the development of AT inflammation. They shift from an anti-inflammatory M2-like (M2-MΦ) to a pro-inflammatory M1-like (M1-MΦ) phenotype and form “crown-like” structures surrounding dead adipocytes (Cinti et al., 2005; Lumeng et al., 2007a; Chawla et al., 2011; Cinti and Giordano, 2020). M1-MΦ secrete pro-inflammatory mediators such as tumor necrosis factor (TNF), interleukin 1 beta (IL-1β), and IL-6 (Chawla et al., 2011; Chmelar et al., 2013; Shapouri-Moghaddam et al., 2018). AT inflammation is also featured by the accumulation within the obese AT of several innate and adaptive immune cells including natural killer cells, neutrophils, CD8^+^ cytotoxic- and type l T helper-lymphocytes, which also produce pro-inflammatory factors (Cildir et al., 2013; Chung et al., 2018; Kane and Lynch, 2019).

Studies suggest that various stromal cells, including AT fibroblasts and endothelial cells, create an adipose niche for APs (Jiang et al., 2017; Zhang et al., 2018), while resident and infiltrating immune cells also contribute to the niche formation, especially in the context of obesity-related inflammation (Nawaz et al., 2017). Considering that preadipocytes are plastic cells that respond to both niche and systemic signals (Jeffery et al., 2016), the pro-inflammatory microenvironment of the obese AT might play a critical role in determining the fate of APs. The present review focuses on the current knowledge about AP fate driven by both intracellular and extracellular factors in the context of obesity-related chronic inflammation.

## Adipogenesis

### White Adipogenesis and AT Expansion

The hyperplastic growth of the AT occurs through the process of adipogenesis, namely the highly dynamic transformation of immature fibroblast-like precursor cells into mature lipid-loaded adipocytes (Rosen and MacDougald, 2006). Peroxisome proliferator-activated receptor-γ (PPARγ) and members of the CCAAT-enhancer-binding proteins (C/EBP) family are master-regulators of this process. However, a considerable number of signaling pathways, including Wnt, Notch, Hedgehog, MAPK, and pro- and anti-adipogenic mediators (KLF and GATA transcription factors, cell cycle proteins) regulate the adipogenic conversion (Farmer, 2006; Rosen and MacDougald, 2006; Ghaben and Scherer, 2019). Of note, APs from different AT depots display qualitative and quantitative heterogeneity. For instance, in both mice and humans, APs from subcutaneous fat depots have shown *in vitro* higher growth rates and adipogenic potential compared to those from visceral AT (Permana et al., 2004; Tchkonia et al., 2005; Macotela et al., 2012). Nevertheless, *in vivo* evidence from adult C57BL/6 mice, supports that following HFD-feeding, visceral AT expands through both adipocyte hypertrophy and hyperplasia, while subcutaneous AT almost exclusively via cellular hypertrophy (Vishvanath and Gupta, 2019). Several markers, such as CD36 and Zfp423 have been suggested to define preadipocyte populations with pronounced adipogenic capacity (Gupta et al., 2012; Gao et al., 2017).

A crosstalk between APs and immune cells could orchestrate adipogenesis in both lean and obese state (Bing, 2015; Chung et al., 2017). For instance, macrophages constitute 5–10% of the SVF in lean mice and their numbers increase in obesity (Weisberg et al., 2003; Weinstock et al., 2019; Daemen and Schilling, 2020). Both proliferation of tissue-resident macrophages and monocyte infiltration contribute to the expansion of this population in the obese AT (Weisberg et al., 2003; Amano et al., 2014; Zheng et al., 2016). Several studies have shown that pro-inflammatory macrophage-conditioned medium inhibits AP differentiation and leads to insulin resistance in mouse and human preadipocytes *in vitro* (Constant et al., 2006; Lacasa et al., 2007; Lumeng et al., 2007b). The potential contribution of individual components of the macrophage secretome to this process has gained strong attention. TNF is a major factor contributing to the anti-adipogenic effect of macrophages, possibly via an epigenetic reprogramming-dependent mechanism (Isakson et al., 2009; Andersen et al., 2019). IL-6 exerts an inhibitory effect on IRS-1, Glut4, and PPARγ, thereby contributing to the decreased adipogenic capacity of human preadipocytes (Rotter et al., 2003; Gustafson and Smith, 2006; Almuraikhy et al., 2016). IL-1β from pro-inflammatory macrophages inhibits insulin sensitivity in both APs and mature adipocytes of mice and humans (Lagathu et al., 2006; Gao et al., 2014). Moreover, the aforementioned cytokines participate in a positive-feedback loop induction of pro-inflammatory gene expression (IL-6, MCP-1, IL-1β, TNF, and IL-8) in APs (Gustafson and Smith, 2006; Isakson et al., 2009; Gao et al., 2014). Priming of human preadipocytes toward a pro-inflammatory phenotype is also mediated by elevated extracellular glucose levels, which accompany obesity and insulin resistance (Rønningen et al., 2015). Pro-inflammatory priming of murine preadipocytes is also mediated by leptin, the levels of which are elevated during obesity (Ouchi et al., 2011; Palhinha et al., 2019). Contrastingly, adiponectin, which is decreased in the obese AT, promotes the differentiation of 3T3-L1 preadipocytes and insulin sensitivity (Fu et al., 2005).

Besides macrophages that contribute to obese AT remodeling, other immune cells within the AT can affect preadipocyte differentiation as well. For instance, murine monocyte-derived dendritic cells (DCs), which accumulate in the obese AT, display anti-adipogenic properties *in vitro* (Pamir et al., 2015; Macdougall and Longhi, 2019). The inhibition of AP differentiation during obesity and AT inflammation promotes the hypertrophic, rather than hyperplastic expansion of the AT, due to storage of the supplied energy in the form of lipids by mature adipocytes and not by differentiating APs (Hammarstedt et al., 2018; Gustafson et al., 2019).

Apart from immune cells, the interaction of APs with the highly abundant endothelial cells of the AT may shape the adipogenic potential of preadipocytes, likely via vasculature-derived factors (Cao, 2007). Indeed, vascular endothelial growth factor (VEGF) is considered a key factor coupling adipo- and angiogenesis in the mouse AT and may favor adipogenesis within adipogenic/angiogenic cell clusters (Nishimura et al., 2007; Sun et al., 2012; Breier et al., 2017). Along this line, a recent study suggested a possible role of endothelial cells in the regulation of fatty acid transport and PPARγ activation in human preadipocytes, due to secretion of PPARγ ligands by endothelial cells (Gogg et al., 2019). Moreover, a spatial and functional overlap of CD34^+^ APs with pericytes has been described, which plays a role in the stabilization of the AT vasculature (Traktuev et al., 2008).

Of interest, a subpopulation of CD142^+^ adipogenesis-regulatory cells (Aregs) was recently identified among the stromal cell population of the mouse AT. This unique subtype of precursor cells is increased in the obese AT and can suppress the differentiation of preadipocytes in a paracrine-dependent manner (Schwalie et al., 2018). However, as described above, a later study did not support this finding (Merrick et al., 2019).

### Beige Adipogenesis

Beige adipogenesis is integral to the metabolic homeostasis of WAT. It contributes to insulin sensitivity by upregulating molecular thermogenic signatures of WAT. Specifically, upon adrenergic stimulation or exposure to cold, beige or brite adipocytes can upregulate UCP-1 expression and adopt a thermogenic phenotype, resembling brown adipocytes (Wu et al., 2012; Alexaki and Chavakis, 2016; Wang and Seale, 2016; Shao et al., 2019). However, a non-canonical UCP-1 mechanism can also contribute to the formation of this phenotype (Bertholet et al., 2017; Ikeda et al., 2018). Lineage-tracing studies allowed to distinguish between Myf5^+^ progenitors that can differentiate into brown adipocytes or myocytes, and Myf5^–^ precursors that are committed to white and beige adipocyte differentiation (Gesta et al., 2007; Obregon, 2014; Peirce et al., 2014). Furthermore, apart from the common white-beige progenitor, beige adipocytes can also derive from a transdifferentiation of mature white adipocytes into beige ones (Lee et al., 2012; Rosenwald et al., 2013).

Until now, “beiging” or “browning” of the WAT is considered as metabolically more favorable than “whitening,” and is essentially a feature of the lean WAT, while it is diminished in the course of obesity and the development of AT inflammation (Alexaki and Chavakis, 2016). Indeed, several innate and adaptive immune cell types have been implicated in the regulation of beige adipogenesis in a positive or negative fashion (Lee et al., 2015; Chung et al., 2017). Multiple studies have suggested the beneficial role of cells of type 2 immunity, such as eosinophils and M2-MΦ in the induction and maintenance of beige adipogenesis in mice, especially in the lean WAT (Lee et al., 2013; Qiu et al., 2014; Alexaki and Chavakis, 2016). For instance, the release of IL-4 by eosinophils is required for the maintenance of the alternatively activated M2-MΦ population, which promote induction of beige adipogenesis in mice (Wu et al., 2011; Qiu et al., 2014; Hui et al., 2015). Nevertheless, contradictory data exist pertinent to the mechanism by which M2-MΦ exert their beige adipogenesis-promoting effect on APs and mature adipocytes and whether the latter may depend on the release of catecholamines by the M2-MΦ or not (Qiu et al., 2014; Fischer et al., 2017). In addition, type 2 innate lymphoid cells (ILC2) promote beige adipogenesis in mice, predominantly by propagating the maintenance of eosinophils and M2-MΦ in a IL-5- and IL-13-related manner (Molofsky et al., 2013). Along this line, type 2 cytokines secreted by ILC2s and eosinophils can stimulate beige adipogenesis in murine PDGFRα^+^ APs, thereby triggering signaling via the IL-4Rα present on the latter (Lee et al., 2015). Another mechanism suggests that mouse AT beiging is stimulated by IL-33-mediated activation of ILC2 and the release by them of a methionine-enkephalin peptide (Brestoff et al., 2014). Interestingly, a recent study showed that murine PRDM16-expressing adipocytes, favor a fibrogenic-to-adipogenic transition of APs, thus promoting beige adipogenesis by secreting β-hydroxybutyrate (Wang W. et al., 2019).

In contrast, the development of a pro-inflammatory microenvironment in the obese WAT restricts the potential of APs toward beige adipogenesis. The increased numbers of M1-MΦ and the concomitant release of pro-inflammatory mediators, such as IL-1β and TNF, contribute to the impaired browning of the obese murine WAT and the suppression of UCP-1 expression, likely in a Toll-like receptor 4 (TLR4) and Nod-like receptor 3 (NLRP3) inflammasome-dependent way (Sakamoto et al., 2016; Okla et al., 2018). Additionally, signaling via the IL-18/IL-18R1 system has been reported to impede energy expenditure and mouse AT beiging *in vivo* (Pazos et al., 2015). Interestingly, a major mechanism involved in the diminished beige adipogenesis in mice and humans during obesity-related WAT inflammation relies on the direct integrin-mediated interaction between M1-like macrophages and APs as well as mature adipocytes (Chung et al., 2017). Furthermore, CD8^+^ T cells were also shown to inhibit beiging of the obese murine WAT in an IFN-γ-dependent manner (Moysidou et al., 2018).

## Fibrosis

Fibrosis is considered a pathophysiological consequence of the persistent low-grade inflammation in the WAT in obesity. Myofibroblasts are the major cell type contributing to the extracellular matrix (ECM) deposition in fibrosis of various organs. They can originate from different precursor cells under the effect of transforming growth factor beta 1 (TGF-β1) and platelet-derived growth factor (PDGF) deriving from inflammatory cells (Wynn, 2008; Sun et al., 2013; Marcelin et al., 2019).

TGF-β inhibits human preadipocyte differentiation into mature adipocytes, while it promotes collagen production and cell proliferation, a process that can be controlled by Jak-Stat signaling (Weiner et al., 1989; Keophiphath et al., 2009; Babaei et al., 2018). TGF-β is involved in the generation of Sca-1^–^SMA^+^ITGA5^+^ fibrogenic progenitor cells in the murine WAT. This process depends on myocardin-related transcription factor A (MRTFA) and results in the shift of the fate of perivascular progenitors from APs with adipogenic potential toward pro-fibrotic cells (Lin et al., 2018).

PDGFRα has been recognized as an anti-adipogenic factor that favors the generation of profibrotic cells in mice (Iwayama et al., 2015; Sun et al., 2017). Marcelin et al. showed the existence of two subsets of PDGFRα^+^ adipocyte progenitors based on the level of their CD9 expression. In both humans and mice, AT CD9^high^ cells were described as pro-fibrotic progenitors, while CD9^low^ precursors were rather committed to adipogenesis. The CD9^low^ subpopulation was almost lost in the fibrotic obese WAT, while CD9^high^ progenitors’ frequency positively correlated with the degree of WAT fibrosis (Marcelin et al., 2017). Similar characterizations of profibrotic vs. adipogenic cells were identified in humans and mice based on Ly6C and CD34 expression. Specifically, Hepler et al. (2018) described in the mouse AT the coexistence of Ly6C^+^ PDGFRβ^+^ fibro-inflammatory progenitors along with the highly adipogenic Ly6C^–^CD9^–^PDGFRβ^+^ cells. Similarly, CD34^high^ APs were described as pro-fibrotic cells in the human visceral AT according to their secretome profile (Buffolo et al., 2019). On the contrary, another study did not find significant differences in proliferative, adipogenic and fibrogenic potential between CD34^–^, CD34^low^, and CD34^high^ cells (Raajendiran et al., 2019). Subpopulations of fibro-inflammatory progenitors increase in numbers following AT expansion, and exert an anti-adipogenic effect on other adipocyte precursor cells via secretion of soluble factors (Marcelin et al., 2017; Hepler et al., 2018; Buffolo et al., 2019).

Importantly, the extent of AT fibrosis positively correlates with the number of crown-like structures in the obese AT (Cinti et al., 2005; Buechler et al., 2015), implying that AT fibrosis may be triggered by the pro-inflammatory microenvironment. Transcriptomic analysis of human preadipocytes cultured with conditioned medium from pro-inflammatory macrophages revealed an upregulation in the expression of ECM components (Henegar et al., 2008). Moreover, macrophage-derived IL-1β promotes the expression of ECM remodeling enzymes, such as metalloproteinases 1 (MMP1) and 3 (MMP3), in human APs (Gao and Bing, 2011). Not only cells of the monocytic lineage contribute to the stimulation of ECM production by preadipocytes. Mast cells accumulate in the mouse and human obese AT preferentially in depots with progressed fibrosis and provoke the secretion of collagen V by AT fibroblasts, which can contribute to the suppression of the adipogenic differentiation of APs. Of note, the secretion of collagen V in the obese AT is triggered by the release of mast cell protease 6 (MCP-6) by mature mast cells (Divoux et al., 2012; Hirai et al., 2014). Other ECM components of the AT, like collagen VI and its derivative endotrophin, can trigger fibrosis in the AT and contribute to preadipocytes’ myofibroblastic transformation (Khan et al., 2009; Sun et al., 2014; Jones et al., 2020). Additionally, while ILC2 drive beige adipogenesis, type 1 innate lymphoid cells (ILC1) promote AT fibrogenesis in human and mice in an IFN-γ dependent manner (Wang H. et al., 2019).

In conclusion, in the lean WAT, APs represent a highly heterogeneous cell population; yet with intrinsic white or beige differentiation potential rather than a pro-fibrotic one. Contrastingly, in obesity, interactions of APs with cells of both the innate and adaptive immunity that accumulate in the obese WAT can trigger fibrosis by inducing a pro-fibrotic transcriptional program in APs.

## Conclusion and Future Perspective

APs are a highly heterogeneous population of stromal AT cells. Different subtypes of APs can have a varying degree of commitment toward white, beige adipocyte or fibroblast differentiation. Along this line, extensive *in vivo* and *in vitro* studies report the identification of numerous AP subpopulations. However, in several of these studies the characterization of the multiple AP subtypes is based on different experimental approaches (Burl et al., 2018; Cho et al., 2019; Min et al., 2019). This issue is further complicated by the regional variation of APs within the different fat depots and the distinct abundance of different progenitor subtypes therein. Thus, the identification of reliable and broadly acceptable molecular and surface markers to distinguish the various AP subtypes is imperative. It is recognized, that the number of adipocytes is set during childhood and adolescence and stays nearly constant in adulthood with a 10% turnover rate in lean and obese individuals (Spalding et al., 2008; Rodríguez et al., 2015; Meln et al., 2019). Consequently, a deeper insight into AP subtypes and crosstalk mechanisms with other cells could shed the light on how the fate of preadipocytes can be predetermined early in life and lead to the development of obesity and accompanying metabolic complications later.

**FIGURE 1:**
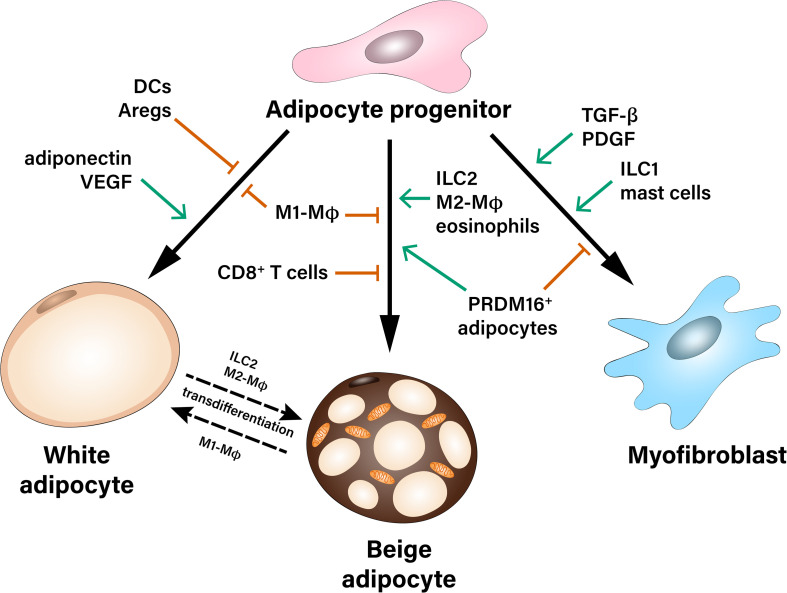
Multiple contact- and paracrine-mediated interactions shape differentiation of adipocyte progenitors toward white or beige adipocytes or myofibroblasts. Pro-inflammatory M1-like macrophages (M1-MΦ) inhibit both white and beige differentiation of preadipocytes. Dendritic cells (DCs), as well as adipogenesis-regulatory cells (Aregs) contribute to the inhibition of white adipogenic differentiation. On the other hand, paracrine factors like adiponectin and VEGF stimulate precursor’s commitment toward white fat cells. Beige adipocytes can derive either from common white/beige adipocyte progenitors or from transdifferentiation of mature white adipocytes. Pro-inflammatory M1-MΦ and CD8^+^ T cells inhibit beige adipogenesis during obesity, while Type 2 innate lymphoid cells (ILC2), M2-like macrophages (M2-MΦ), eosinophils as well as a subpopulation of PRDM16^+^ adipocytes were described as positive regulators of beige adipogenesis. Adipocyte progenitors can differentiate into myofibroblasts and therefore, contribute to the development of fibrosis. This process is stimulated by pro-fibrotic factors (TGF-β, PDGF) as well as type 1 innate lymphoid cells (ILC1) and mast cells.

Importantly, the crosstalk between APs and immune cells in the AT orchestrates AP fate in both lean and obese state. For instance, obesity-related AT inflammation leads to reduced beige adipogenic and increased pro-fibrotic potential of APs. So far, the majority of studies have focused on the interaction between macrophages and APs, while less information exists pertinent to the role of cells of the adaptive immunity as well as less abundant stromal cell types, which may also shape the differentiation potential of APs (Figure 1). Identification of the specific contribution of different immune and stromal cell populations, which may affect fate decisions of APs, as well as better understanding of the molecular mechanisms implicated in this crosstalk is needed for the development of new therapeutic strategies against obesity-related AT dysfunction.

## Author Contributions

IP, TC, and AC wrote the manuscript, while ZM, K-JC, and MK edited the manuscript. All authors contributed to the article and approved the submitted version.

## Conflict of Interest

The authors declare that the research was conducted in the absence of any commercial or financial relationships that could be construed as a potential conflict of interest.
